# Effects of *Hypericum scabrum* extract on dentate gyrus synaptic plasticity in high fat diet-fed rats

**DOI:** 10.1186/s12576-020-00747-0

**Published:** 2020-03-24

**Authors:** Ghazaleh Omidi, Arezoo Rezvani-Kamran, Ahmad Ganji, Somayeh Komaki, Farshid Etaee, Masoumeh Asadbegi, Alireza Komaki

**Affiliations:** 1 Neurophysiology Research Center, Hamadan University of Medical Sciences, Hamadan, Iran; 2 Rahe Sabz Addiction Rehabilitation Clinic, Hamadan University of Medical Sciences, Hamadan, Iran; 3 Department of Physiology, School of Medicine, Hamadan University of Medical Sciences, Shahid Fahmideh Street, 65178/518 Hamadan, Iran

**Keywords:** Synaptic plasticity, *Hypericum scabrum*, High-fat diet, Long-term potentiation antioxidant

## Abstract

High-fat diet (HFD) can induce deficits in neural function, oxidative stress, and decrease hippocampal neurogenesis. *Hypericum* (*H.*)* scabrum* extract (Ext) contains compounds that could treat neurological disorders. This study aimed to examine the neuroprotective impacts of the *H. scabrum* Ext on hippocampal synaptic plasticity in rats that were fed HFD. Fifty-four male Wistar rats (220 ± 10 g) were randomly arranged in six groups: (1) HFD group; (2) HFD + Ext300 group; (3) HFD + Ext100 group; (4) Control group; (5) Ext 300 mg/kg group; (6) Ext 100 mg/kg group. These protocols were administrated for 3 months. After this stage, a stimulating electrode was implanted in the perforant pathway (PP), and a bipolar recording electrode was embedded into the dentate gyrus (DG). Long-term potentiation (LTP) was provoked by high-frequency stimulation (HFS) of the PP. Field excitatory postsynaptic potentials (EPSP) and population spikes (PS) were recorded at 5, 30, and 60 min after HFS. The HFD group exhibited a large and significant decrease in their PS amplitude and EPSP slope as compared to the control and extract groups. In reverse, *H. scabrum* administration in the HFD + Ext rats reversed the effect of HFD on the PS amplitude and EPSP slope. The results of the study support that *H. scabrum* Ext can inhibit diminished synaptic plasticity caused by the HFD. These effects are probably due to the extreme antioxidant impacts of the Ext and its capability to scavenge free radicals.

## Introduction

Modifications in diet and lifestyle, which may have happened with industrialization, urbanization, economic development, and market globalization have occurred previously [1]. It is assumed that lifestyle plays an essential role in maintaining neural function [2]. The continuous and long-term consumption of a high-fat diet (HFD) leads to weight gain and obesity [3]. HFD is the most significant risk factor for diseases related to lifestyle [4].

The increased incidence of obesity and obesity-associated comorbidities is a global health concern [4]. Although the adverse effects of obesity in the brain are unclear, studies have suggested that obesity and body fat deposition play an essential role in the pathogenesis of certain brain-related disorders [3, 5, 6]. Recent studies have also demonstrated that HFD consumption and obesity are correlated with cognitive damage and a raised chance of expanding dementia [7, 8].

In addition, recent data suggest that acute consumption of HFD can lead to memory deficits and significant brain inflammation [9]. Obesity is also linked to apparent oxidative stress and chronic inflammatory status. Oxidative stress along with a decline in antioxidant defenses cause irreversible damage to macromolecules [10] and disruption in redox signaling mechanisms [11, 12].

An HFD reduces molecules related to learning and memory, such as levels of brain-derived neurotrophic factor (BDNF), and dopamine (DA) [13]. In animal studies, HFD has been shown to impair hippocampal neurogenesis and specifically, synaptic plasticity in the dentate gyrus [14–16]. HFD can decrease hippocampal neurogenesis and lead to oxidative stress by provoking lipid peroxidation in the hippocampus [16]. Therefore, it has been reported that an HFD causes cellular injury by inducing oxidative stress [17, 18]. Additionally, HFD elevates the levels of neuroinflammation markers in the brain [19] and it might cause degenerative disorders via insulin resistance [20, 21].

The reactive oxygen species (ROS) have an impact on various physiological activities. Nevertheless, when ROS concentration exceeds the antioxidative capability of an organism, it causes oxidative damage to cellular elements [22]. Previous studies have shown the role of oxidative damage in memory deficits in rats and humans [23]. The brain is susceptible to oxidative stress because of its high amount of polyunsaturated fatty acids (lipid peroxidation), high oxygen utilization, low levels of antioxidant protection, and the presence of redox-active metals (Fe, Cu) [24]. Previous studies have emphasized the importance of antioxidant treatment in the prevention of oxidative stress-induced neuronal injury [25].

Herbs possess some of the most potent natural antioxidants such as phenols, phenolic elements, or flavonoids [26]. Recent studies have indicated promising effects of herbal medicines in the treatment of various memory disorders [27–29]. These effects could correlate to their antioxidant and anti-inflammatory properties [30–32].

*Hypericum* (*Hypericaceae*) (*H.*) genus includes over 400 species and is distributed all over the world. It is well-distributed over subtropical regions and tropical, as well as across Africa, Asia, Europe, and North America [33, 34]. The therapeutic effect of *Hypericum* (*H.*) species is related to the presence of various bioactive compounds, including flavonoids, phloroglucinols, naphthodianthrones, phenolic acids and also essential oil [35, 36]. *Hypericum* (*H.*)* scabrum* extract (Ext) contains flavonoids such as quercitrin and quercetin that exhibit free radical scavenging action. The antioxidant activity of quercetin was confirmed by the inhibition of lipid peroxidation [37]. Clinical effects of *Hypericum* include amelioration of neurological disorders, anti-anxiety, antidepressant, antioxidant, anti-inflammatory, anticonvulsant, antiviral, antifungal, and wound healing [34, 35, 38, 39]. *H. scabrum* essential oil may be helpful in treating the central nervous system disorders [35].

Long-term potentiation (LTP) is a kind of synaptic plasticity, which is established as a long-lasting augmentation of synaptic communications [40]. This phenomenon in the hippocampus and elsewhere is a probable synaptic substrate of the long-term learning and memory modifications [41]. The dentate gyrus (DG) is part of the hippocampus which is thought to contribute to process of learning and memory through activity of dentate granule neurons [42]. This part is one of the few areas of the rat brain which continues to produce new neurons well after birth [43, 44]. In the DG, electrical stimuli delivered to perforant pathway (PP) evoke field excitatory postsynaptic potential (EPSP) [45, 46]. Hence, we hypothesized that the application of *H. scabrum* Ext would ameliorate HFD-induced synaptic plasticity impairment due to its antioxidant and anti-obesity impacts. In this investigation, we assessed the impacts of *H. scabrum* Ext on LTP impairment induced by HFD.

## Methods

### Animals

**Fig. 1:**
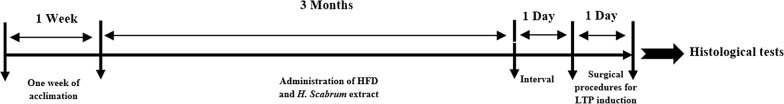
Experimental timeline. Following 3 months of high-fat diet (HFD) and administration of *H. Scabrum* extract in the experimental groups, with an intraperitoneal urethane rats were anesthetized and then put in a stereotaxic apparatus for surgery and electrophysiological recording. At least 20 min of stable baseline response was taken and then, using a high-frequency stimulation protocol, long-term potentiation (LTP) was recorded

The researchers purchased adult male Wistar rats from Pasteur Institute. All animals were accommodated in a place maintained at a steady temperature (22 ± 2 °C), 60 ± 5% humidity and a 12-h light–12-h dark cycle. Rats were provided unrestricted access to water and rodent chow. Rats were acclimatized to regular rodent food for 1 week. Fifty-four rats (weighing 220 ± 10 g) were divided into 6 groups (*n* = 9): (1) HFD group, given an HFD with 45% energy from fat; (2) HFD + Ext300 group, given an HFD supplemented with *H. scabrum* extract (300 mg/kg); (3) HFD + Ext100 group, given an HFD supplemented with *H. scabrum* extract (100 mg/kg); (4) control group, given a normal diet with 10% energy from fat and received the saline through oral gavage once a day for 3 months; (5) *H. scabrum* 300 mg/kg (Ext300) group, given the standard diet supplemented with *H. scabrum* (300 mg/kg); (6) *H. scabrum* 100 mg/kg (Ext100) group, given the standard diet supplemented with *H. scabrum* (100 mg/kg). Rats received the extract through oral gavage once a day for 3 months [3, 14, 27]. The gavage was performed from 9:00 to 11:00 a.m. All experimental procedures were done under international standards of animal care approved by the Society for Neuroscience. The experimental timeline is shown in Fig. 1.

### *Hypericum scabrum* extract

The plant was extracted with 70% ethanol; then evaporated. The evaporation process included the total removal of ethanol and water [47]. *H. scabrum* extract at doses of 100 and 300 mg/kg was given by gavage in the 2 groups that were taking normal diet and in the 2 groups that were taking HFD daily for 3 months. These doses were determined based on former studies [3, 27].

### Surgical procedures for LTP induction

The methodology used in this section was similar to previous studies published by our laboratory [48, 49]. Under the anesthesia induced by intraperitoneal injection (1.5 g/kg) of urethane, rats’ heads were fixed in a stereotaxic apparatus for surgery and recording. The rats’ temperature were maintained at 37.0 ± 0.2 °C with an electrical warming pad during the operation. A bipolar stimulating wire electrode, made of stainless-steel with Teflon cover (125 μm inner diameter/175 μm external diameter, Advent Co., UK), was inserted into the PP (3.2 mm ventral below the surface of the skull, 4.3 mm lateral to the midline, 8.1 mm posterior to bregma), in accordance with the atlas of Paxinos and Watson [50]. Moreover, a bipolar recording electrode (2.3 mm lateral to the midline, 3.8 mm posterior to bregma) was lowered into the DG until the maximal field EPSP were recorded (2.7–3.2 mm ventral). The optimal ventral location was obtained by electrophysiological monitoring of the result provoked in the DG following PP stimulation [51, 52].

### High-frequency stimulation for LTP induction

Input/output (I/O) response curve was created via different intensities of the single pulse stimulation (0.1 ms biphasic square wave pulses at a frequency of 0.1 Hz) [49, 52]. Afterward, single stimuli was utilized every 10 s for at least 30 min, and results were monitored. LTP was provoked by using a 400 Hz (10 bursts of 20 stimuli, 0.2 ms stimulus duration (biphasic square wave pulses), 10 s inter-burst interval) high-frequency stimulation (HFS) protocol at a stimulus intensity that elicited a population spike (PS) amplitude and the field EPSP slope of nearly 50% of the maximal response. After HFS, EPSP and PS were registered at 5, 30, and 60 min for assigned alterations into the synaptic response of the DG neurons. For every time point, ten sequential provoked results were averaged at 10 s stimulus intervals [53, 54]. The single pulses of the post-LTP burst were the same type as those for the I/O curve. A single LTP test was conducted per animal.

### Histology

**Fig. 2:**
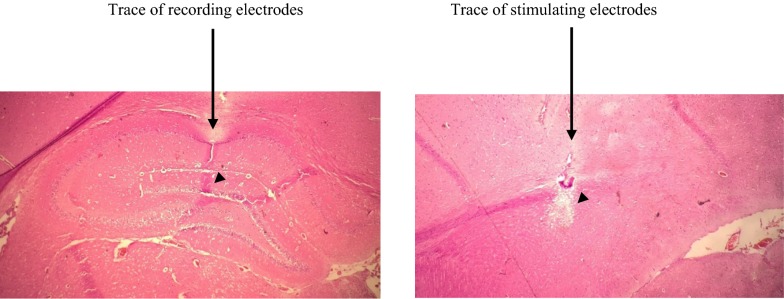
The positions of stimulating and recording electrode tips (arrowheads) are shown in a photomicrograph from a coronal hippocampus section. Electrode traces of the stimulating and recording sites are exposed in both sides (arrows). Scale bar: 0.5 mm

Following the fulfillment of the investigation, the electrode sites in the hippocampus were defined histologically. After the conclusion of the experiments, rats were completely anesthetized with urethane, and formal-saline was infused via the heart [55]. Coronal brain sections were cut at 50 μm and stained with hematoxylin–eosin for histological corroboration and confirmation of electrode tip location [51] (Fig. 2).

### Statistical analysis

We used the GraphPad Prism version 5.00 (GraphPad Software, San Diego, California USA) for data analysis. The data are demonstrated as the mean ± standard error of the mean (SEM). Two-way repeated measures analysis of variance tests was applied to statistically analyze the results. The Tukey test was used for post hoc comparisons between experimental groups. Statistical differences were considered significant at *P* < 0.05.

## Results

### Measurement of evoked responses

**Fig. 3:**
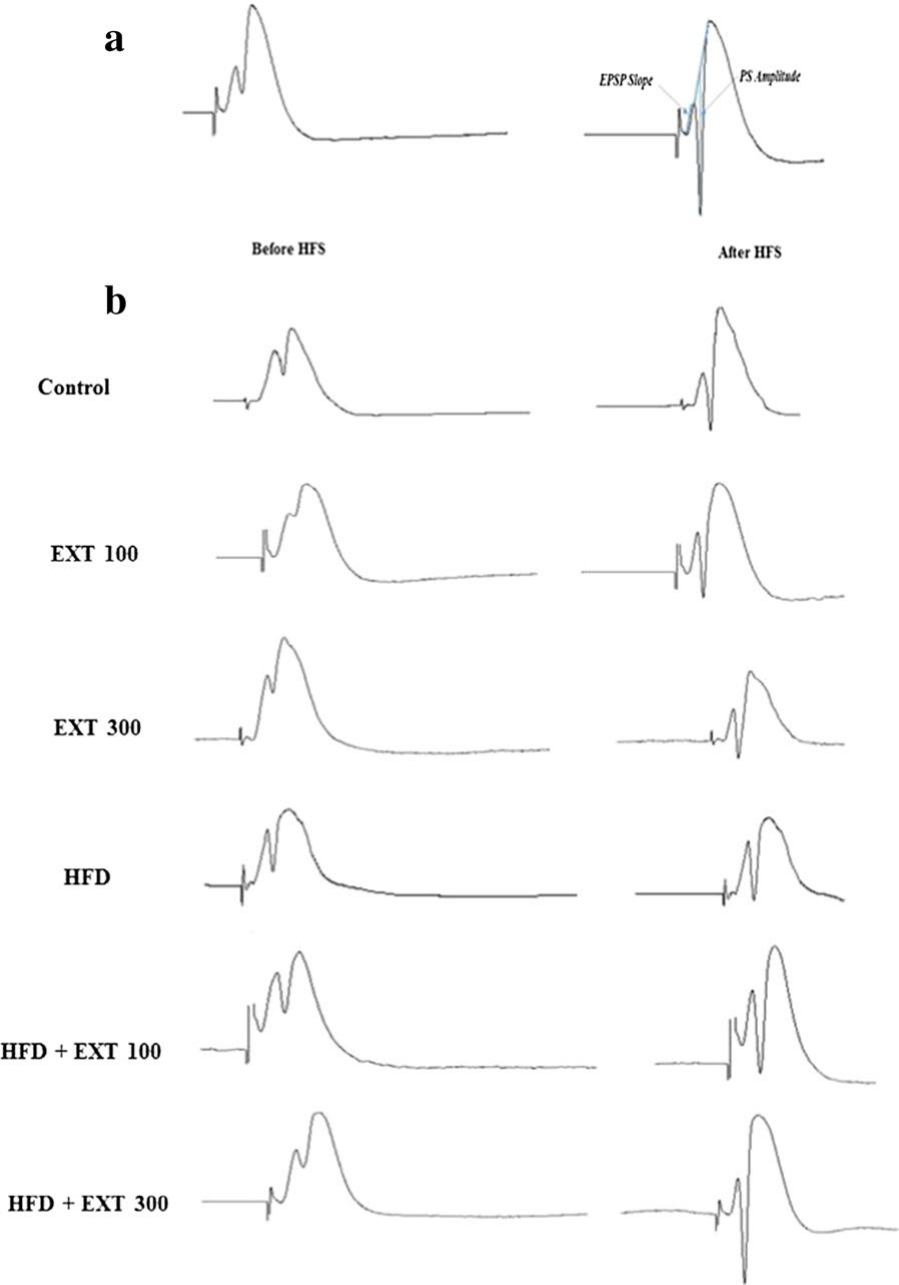
Measurement of evoked responses. Changes in excitatory postsynaptic potential (EPSP) slope and population spike (PS) amplitude in the perforant pathway–dentate gyrus (PP–DG) synapses were recorded following high-frequency stimulation (HFS). The arrows indicate PS and the slope of the EPSP (**a**). Typical evoked field potential sample traces in the DG recorded before and 60 min after HFS in all experimental groups (**b**)

The DG responses including, EPSP and PS were recorded after stimulation of the PP (Fig. 3). Changes in EPSP slope and PS amplitude were recorded throughout the electrophysiological recording. The amplitude of the PS was determined from the peak of the first positive deflection of the evoked potential to the peak of the next negative potential. Using an input/output curve stimulation intensity was adapted to provoke potentials, including 40% of the maximal PS amplitude. Then, using a data analysis software, slopes for EPSPs and PS amplitude were calculated.

### Effects of *H. scabrum* extract supplementation on the EPSP slopes of granular cells in the DG of rats fed with HFD

**Fig. 4:**
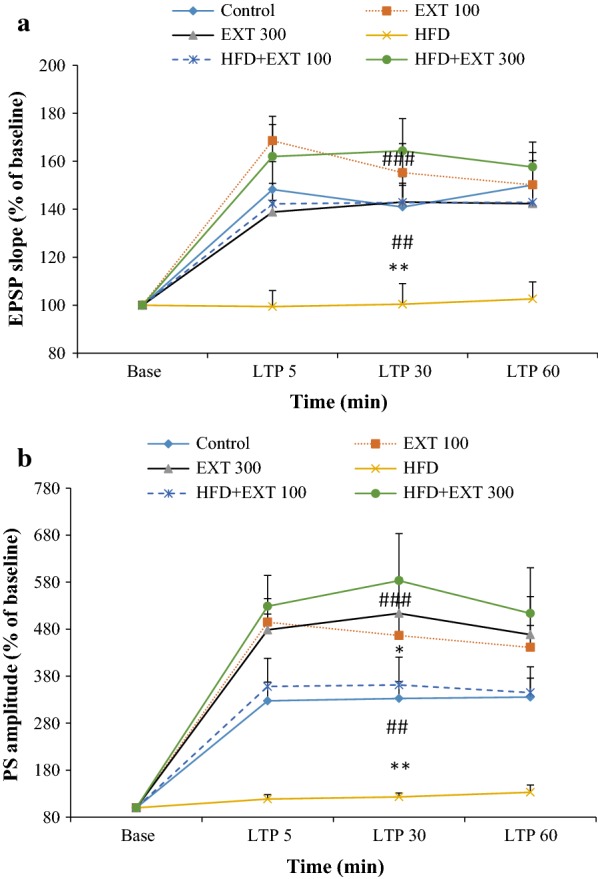
Time-dependent changes of long-term potentiation (LTP) of the excitatory postsynaptic potentials (EPSP) slope (**a**) and population spike (PS) (**b**) amplitudes in dentate gyrus (DG) granular cell synapses in response to perforant pathway (PP) stimulation after a high-frequency stimulation (HFS). Data are shown as means ± SEM % of baseline. *P < 0.05; **P < 0.01 (compared with control); ^##^*P* < 0.01; ^###^*P* < 0.001 (compared with HFD)

Field potential recordings were obtained in the granular cells in the DG following stimulation of the PP. HFS of the PP caused LTP in the DG. The effects of *H. scabrum* extract on the EPSP slopes and PS amplitudes of the HFD-fed rats are shown in Fig. 4a, b, respectively. The effects of dietary supplementation with *H. scabrum* extract on the LTP of EPSP induced by HFS in the PP to the DG area of the hippocampus in rats were examined.

We used a two-way ANOVA to reveal the variability between the groups. Our results showed a significant effect of time-points [*F* (3, 192) = 35.47, *P* < 0.0001], treatment [*F* (5, 192) = 14.26, *t* < 0.0001] and interaction (time * treatment) [*F* (15, 192) = 1.808, *P* = 0.0359] in slope of EPSP of the granular cell of DG (Fig. 4a). Our post hoc analysis indicated significant differences between the control group and the HFD animals (*P* < 0.01, Fig. 4a). Slope of EPSP decreased in the HFD group with respect to other groups (HFD: 100.83 ± 7.46; control: 153.88 ± 16.55; Ext100: 153.02 ± 18.28; Ext300: 158.89 ± 21.95; HFD + Ext100: 142.65 ± 7.73; HFD + Ext300: 161.3067 ± 13.35). These values represent the average of responses in 5, 30 and 60 min after HFS in each group. *H. scabrum* extract supplementation in HFD animals significantly compensated the decrease in the EPSP slope in the HFD group (*P* < 0.05, Fig. 4a). Extract administration significantly compensated the decrease in the EPSP slope compared with the HFD group (HFD + Ext100 group: *P* < 0.01; HFD + Ext300 group: *P* < 0.001, Fig. 4a). There were no significant differences between control and the remaining groups (Fig. 4a).

### Effects of *H. scabrum* extract supplementation on the PS amplitude of granular cells in the DG of rats fed with HFD

We used a two-way ANOVA to reveal the variability between the groups. Our results showed a significant effect of time-points [*F* (3, 192) = 61.15, *P* < 0.0001], treatment [*F* (5, 192) = 25.51, *P* < 0.0001] and interaction (time * treatment) [*F* (15, 192) = 2.962, *P* = 0.0003] in PS amplitude of the granular cell of DG (Fig. 4b). Our post hoc analysis indicated significant differences between the control group and the HFD animals (*P* < 0.01, Fig. 4b). PS amplitude decreased in the HFD group with respect to control group (HFD: 124.88 ± 11.00; control: 331.8 ± 38.58; Ext100: 467.45 ± 71.52; Ext300: 459.53 ± 63.18; HFD + Ext100: 354.70 ± 57.95; HFD + Ext300: 541.73 ± 87.58). *H. scabrum* extract administration in HFD-fed animals significantly compensated the decrease in the PS amplitude in the Aβ group (*P* < 0.001, Fig. 4b). In addition, Ext supplementation in control animals enhanced PS amplitude of the granular cell of DG (Fig. 4b). Extract significantly augmented the PS amplitude compared with the HFD group (HFD + Ext100: *P* < 0.01; HFD + Ext300; *P* < 0.001). There were no significant differences between Ext100, Ext300, and control groups (Fig. 4b).

## Discussion

This investigation appraised the effect of the application of the hydroalcoholic extract of *H. scabrum* on synaptic plasticity using the field potential response in HFD-treated rats. The results showed that HFD reduced EPSP slope and PS amplitude in the HFD groups and *H. scabrum* application was able to neutralize the negative effects of HFD on-field potential recordings. Our findings showed that *H. scabrum* extract ameliorates HFD-induced synaptic plasticity deficits, as measured by an augmentation in LTP of granular cells in the DG after stimulation of the PP. It should be noted that only a single LTP was induced in each animal. The induced LTP was compared between experimental groups.

Oxidative stress has been proven to play an essential function in cognitive damage [56]. A balance between free radical production and antioxidant capacity is critical, and oxidative stress results from the accumulation of oxidative products [57]. The excess formation of oxidants can cause oxidative stress [3, 58, 59]. A HFD increases plasma-free fatty acids and induces oxidative stress from the accumulation of lipid peroxidation in the hippocampus [60]. It has been reported that oxidative stress causes neurodegenerative diseases such as AD [61]. Mitochondrial injury and HFD-evoked oxidative stress are reasons that could conduce to some forms of neurodegeneration [62]. Furthermore, recent investigations have proposed the use of antioxidant supplementation to decrease oxidative stress-induced neurodegeneration [63, 64]. Some investigations have remarked that oxidative stress causes neural injury in brain regions associated with the etiology of memory damage [65–67]. Furthermore, oxidative stress could influence synaptic plasticity and can lead to diminished LTP induction [40]. In contrast, some of the previous studies have shown that antioxidants improve the induction of hippocampal LTP [68]. An important new finding of this study was that the adverse effects of the HFD were reduced substantially by the administration of the dietary *H. scabrum* extract. In our study, Ext administration significantly compensated the decrease in the PS amplitude and EPSP slope compared with the HFD group. All of the parts of this plant are sources of fatty acids, especially essential fatty acids, as well as effective natural antioxidants [69]. It has been indicated that, its extract possess flavonoids, such as quercetin [70]; flavonoids act as antioxidants [71]. In confirmation of our results, administration of *H. scabrum* Ext in rats was shown to improve learning and memory and possess antioxidant activity [27]. In accordance with this finding, Pintana et al. [72] have shown that an HFD impairs learning and memory, and treatment with garlic extract restores these impairments in obese insulin-resistant rats. In our previous investigation [3], the rats in the HFD groups demonstrated a significant reduction in glutathione level in comparison to those in extract and control groups, whereas, the malondialdehyde levels in the HFD groups were significantly greater than those in the extract and control groups. The different investigations discovered that *H. scabrum* protects DNA against oxidative injury via its significant antioxidant effects [73].

HFD-induced obesity and increase in inflammatory markers in animals are connected [74]. Accordingly, a long-term HFD (16 weeks) has been shown to cause anxiety, increase corticosterone level, and increase inflammatory cytokines, such as interleukin-6, interleukin-1β, and tumor necrosis factor-α [75]. In contrast, *H. scabrum* extracts have been reported to inhibit both lipoxygenase and cyclooxygenase pathways, which then reduces inflammatory factors [76, 77]. Additionally, it has been reported that, the *H. scabrum* extract has anti-inflammatory properties [78] that probably result from the flavonoids in the extract [79].

Our investigation indicated that PS amplitude and EPSP slope decreased in the HFD group compared to the control group. Our findings are corroborated by numerous investigations that indicated the negative result of HFD on learning and memory. Consistent with our results, it has been published that HFD can diminish cognitive effects [80] and synaptic plasticity [68]. In accordance with our results, male rats on an HFD exhibit reduced hippocampal neurogenesis [81]. Moreover, HFD compromises the expression of a number of neurotrophic factors that would enhance hippocampal plasticity [2, 82]. Moreover, previous investigations have suggested a link between high-fat intake and cognitive impairment. They found that an HFD reduced hippocampal brain-derived neurotrophic factor (BDNF) levels, neuronal plasticity, and learning in rats [2, 83]. In this line, multiple studies have demonstrated that HFD intake is associated with decreased expression of BDNF in both the hippocampus [84, 85] and cortex [86, 87] suggesting that the adverse effects of HFD consumption on learning and memory may also be mediated in part by alteration of BDNF-related synaptic plasticity [8].

Hypericum displayed some impacts directly related to brain activity, such as repression of the neuronal reuptake of norepinephrine, 5-HT, l-glutamate, GABA, and DA and augmented receptor binding and neurotransmitter sensitivity [88]. It has been reported that, Hypericum extract has functional impacts, including amelioration of cognitive impairment, neuroprotective effects, and improvement of cognitive performance in rodents [89].

## Conclusion

In conclusion, the current investigation indicates that *H. scabrum* extract treatment can prevent synaptic plasticity impairment caused by HFD. These effects may be a result of the strong anti-inflammatory and antioxidant activity of the extract and its capability to scavenge free radicals. Prospective investigations are required to test further this matter and the precise mechanism underlying the impacts of *H. scabrum* extracts on synaptic plasticity.
